# Hydrozone in Gastric and Intestinal Disorders*Published by the New York Medical Journal, August 15, 1896.

**Published:** 1897-02

**Authors:** John Aulde

**Affiliations:** Philadelphia, Pa.


### HYDROZONE IN GASTRIC AND INTESTINAL DISORDERS.*

BY JOHN AULDE, M. D., PHILADELPHIA, PA.

A period of nearly twelve years has elapsed since I first began the clinical use of hydrogen dioxide, generally referred to at that time as the peroxide of hydrogen. In 1887 I published a paper giving a detailed account of several cases in which it had been employed by inhalation, but even then I was thirty years behind the report of Dr. (now Sir) Benjamin Ward Richardson, of London, who had made a thorough investigation of its antiseptic, detergent, and healing properties. Notwithstanding the fact that this preparation had been known to the medical profession for that length of time it had achieved little or no reputation. This, however, may be explained by the fact that the discovery preceded the dawn of bacteriology. Indeed, I was one of the early contributors to medical literature relating to the clinical value of this product, and since that time I have published a number of articles, embracing practically every application, both medical and surgical, to which hydrogen dioxide is adapted.

In the present communication it is my object to direct the attention of the profession to its special value in the treatment of gastric and intestinal disorders. In gastritis, for example, there is no antiseptic which can be given with so much benefit as this remedy, because its effect is immediate, and even in considerable doses it is absolutely harmless. The same is true in regard to its employment in typhoid fever, cholera infantum, and Asiatic cholera. In the latter disease its efficacy has been thoroughly demonstrated by a number of well-known physicians, and its applicability in cholera infantum is well known to those physicians who have given careful attention to the most modern methods in the treatment of this class of cases.

The following brief notes will be sufficient to indicate the availability of this remedy in the treatment of the disorders already mentioned, although, in view of the fact that hydrozone is a more concentrated product, and withal a permanent

* Published by the New York Medical Journal, August 15, 1896.

solution, this latter remedy should have the preference. It contains at least double the volume of nascent oxygen which has heretofore been the standard for the medicinal peroxide of hydrogen.

In gastritis, either acute, subacute, or chronic, we have to deal with an unhealthy condition of the lining membrane of the stomach. The inflammation is attended with an increased output of mucus, which seriously interferes with the normal functions of the peptic glands. By the introduction of a small quantity of hydrozone, in the strength of one part to thirty-two parts boiled or sterilized water, this objectionable mucus is at once destroyed by the action of the oxygen which is released, and the contents of the stomach remaining are promptly discharged into the small intestine. A patient suffering from gastritis should take at least half an hour before meals from two to four ounces of diluted hydrozone (one to thirty-two) and lie on the right side, so as to facilitate the action of the stomach in discharging its contents.* The antiseptic proper ties of hydrozone thus used are sufficient to destroy the micro-organisms and leave the stomach in a healthy condition for the absorption of nutritive pabulum. All forms of fermentation are promptly subdued by the active oxidation resulting from the liberation of nascent oxygen. The patient is then in a condition to take suitable food, which should be nutritious and easily digested, liquids being preferred until the active symptoms have subsided. Later, small portions of solid food can be ingested, but all food stuffs of a starchy character must be thoroughly masticated, in order to secure the action of the salivary secretion upon the starch granules, breaking them up, and lessening the tendency to fermentation in the stomach. After taking a meal, a patient with gastritis should follow it with medicinal doses of glycozone, which contain, in addition to the nascent oxygen contained in hydrozone, a percentage of glycerin, which favors osmosis and assists in re-establishing the functional activity of both the peptic and mucus glands of the organ.

In the treatment of cholera infantum, typhoid fever and Asiatic cholera, the same general plan should be adopted in dealing with the stomach, always bearing in mind the necessity for having the patient remain in the recumbent position and on the right side for at least half an hour after the ingestion of the solution. In addition, however, to the preliminary treatment of the stomach, the same solution (one to thirty-two) is used as an injection into the lower bowel, care being exercised to insure its introduction as high up as possible. This

*In chronic cases with a large output of gastric mucus, and particularly in gastric ulcer, concentrated solutions are not well borne at first, owing to the formation of oxygen gas, but this difficulty disappears with the continued use of the remedy, and no treatment of gastric ulcer can be regarded as complete without the local employment of hydrozone.

can be managed by having the patient lie on the left side, with the hips well elevated, and the employment of a long, flexible rectal tube. In this manner we secure and maintain an antiseptic condition in both the stomach and large intestine, the importance of which will be understood when we consider the large number of micro-organisms which grow under these favorable conditions with such remarkable rapidity.

When deemed advisable, the solution introduced into the lower bowel may be combined with large quantities of either hot or cold water, which enables us to obtain the benefits of irrigation in addition to the antiseptic effects. These irrigations may be employed as frequently as deemed advisable by the medical attendant, but they will usually prove satisfactory if administered at intervals of four hours.

Although brief, it is believed this communication will prove serviceable to a large number of practitioners who have hitherto found serious difficulties in counteracting the mephitic influences of bacteria in this class of disorders, and the clinical virtues of the remedy being now so fully recognized, no one will hesitate to adopt the methods suggested, which may be conveniently carried out in addition to the usual routine treatment.

